# Fixational eye movements predict visual sensitivity

**DOI:** 10.1098/rspb.2015.1568

**Published:** 2015-10-22

**Authors:** Chris Scholes, Paul V. McGraw, Marcus Nyström, Neil W. Roach

**Affiliations:** 1 Visual Neuroscience Group, School of Psychology, University of Nottingham, Nottingham NG7 2RD, UK; 2 Humanities Laboratory, Lund University, Helgonabacken 12, 22362 Lund, Sweden

**Keywords:** microsaccades, fixational saccades, contrast sensitivity, machine learning

## Abstract

During steady fixation, observers make small fixational saccades at a rate of around 1–2 per second. Presentation of a visual stimulus triggers a biphasic modulation in fixational saccade rate—an initial inhibition followed by a period of elevated rate and a subsequent return to baseline. Here we show that, during passive viewing, this rate signature is highly sensitive to small changes in stimulus contrast. By training a linear support vector machine to classify trials in which a stimulus is either present or absent, we directly compared the contrast sensitivity of fixational eye movements with individuals' psychophysical judgements. Classification accuracy closely matched psychophysical performance, and predicted individuals' threshold estimates with less bias and overall error than those obtained using specific features of the signature. Performance of the classifier was robust to changes in the training set (novel subjects and/or contrasts) and good prediction accuracy was obtained with a practicable number of trials. Our results indicate a tight coupling between the sensitivity of visual perceptual judgements and fixational eye control mechanisms. This raises the possibility that fixational saccades could provide a novel and objective means of estimating visual contrast sensitivity without the need for observers to make any explicit judgement.

## 1. Introduction

Even during stable visual fixation, when the visual world seems stationary, our eyes are moving. These movements consist mainly of slow drifts in eye position that are punctuated by tiny (<1°) rapid flicks (fixational saccades) at a rate of around 1–2 per second [1]. Fixational saccades share many features with larger voluntary saccades (such as a correlation between amplitude and peak eye velocity [2]) and an increasing body of neurophysiological evidence points to a common generation mechanism involving the superior colliculus (SC) [3,4–8] and cerebellum [9,10] with modulation (at least for voluntary saccades) by cortical regions including the lateral intraparietal area and the frontal eye fields [11–13].

The influence of fixational saccades on visual processing has been investigated at both the physiological and perceptual levels. Firing rates of individual neurons in the SC, lateral geniculate nucleus, V1, V4, MT and intraparietal cortex are suppressed before and during fixational saccades [14–17]. Mirroring findings with large voluntary saccades, a variety of perceptual costs have been reported around the time of fixational saccades, including reduced contrast sensitivity [18–20] (but see [21,22]), impaired motion detection [17] and distortions of perceived position [23]. Recent work, however, suggests that fixational saccades may facilitate the processing of fine spatial detail [24,25] by re-positioning gaze on the most sensitive parts of the fovea [26,27]. They may also serve a functional role in counteracting visual fading in the peripheral visual field [28,29], though this view has recently been criticized on several grounds [27].

As well as exerting an effect on visual processing, the production of fixational saccades is itself shaped by visual input [30–32]. The presentation of a peripheral visual stimulus biases the distribution of fixational saccade directions: they tend to be directed first towards the location of the stimulus and subsequently back towards fixation, a pattern that has been attributed to shifts in covert attention [8,33–37]. Visual input also exerts a modulatory influence on the frequency of fixational saccades. Following the presentation of a visual stimulus, fixational saccade rate exhibits a characteristic biphasic signature: initially decreasing (inhibition) before rebounding to a higher level and then returning to baseline levels [1,38]. This rate signature is induced by a wide variety of stimulus transients, even when the stimulus is task-irrelevant [1,33,38].

To date, studies of the rate signature have opted to aggregate data across multiple subjects and compare fixational saccades across a limited number of discrete conditions. For example, a recent study compared the mean rate signatures in a group of 27 subjects at three different luminance contrast levels, finding systematic changes in the amplitude and latency of the inhibition phase [38]. Here we take a parametric approach to probing the contrast sensitivity of the rate signature in individual subjects. We first show that features of the rate signature change systematically across small increments in contrast close to subjects' detection thresholds. By training a machine-learning algorithm to classify trials on the basis of whether a stimulus was presented, we further demonstrate that fixational saccades during passive viewing can be used to accurately predict individual psychophysical contrast sensitivity.

## 2. Methods

### (a) Participants

Seven participants (two females; mean age = 32, range = 19–46) with normal or corrected-to-normal vision participated in the study. Five observers were naïve to the aim of the experiment, including one observer who had little or no experience of visual psychophysics.

### (b) Stimulus materials and procedure

Observers sat in a dark room and were instructed to maintain fixation on a central white dot (0.08° diameter; Weber contrast 0.95). The head was secured using a chin and forehead rest. Stimuli were large Gabor patches presented centrally (standard deviation of 5°; spatial frequency of 0.33 cycles deg^−1^; 1 frame duration at 85 Hz). Phase was randomized to prevent the build-up of a retinal afterimage and orientation was randomly set to ±45°. There were two trial types: passive and response. During response trials (indicated by a synchronized tone pip), observers were required to indicate the orientation of the Gabor using the left and right arrow keys on a keyboard. During passive trials, no response was required. Passive and response trials were randomly interleaved with inter-trial intervals randomly selected from a uniform distribution (1–1.4 s) to counteract effects of expectation observed when fixed intervals are used [8,34]. We opted to have distinct response and passive trials because the fixational saccade rate is modulated by manual response preparation [39]. However, the two types of trials were interleaved so that any contrast sensitivity differences due to tear break-up [40], learning or fatigue affected oculomotor and behavioural estimates equally. Stimulus contrast was randomly selected from the range 0.7 to 4% (12 contrasts with log steps), with the addition of a baseline condition (0%) for passive trials.

Stimuli were generated using PsychoPy [41,42] on a Viglen computer and presented on an 18 inch CRT monitor (Clinton Monoray, CRS Ltd, Cambridge, England; resolution 1024 × 768; *I*_b_ = 148 cd m^−2^) with a viewing distance of 65.5 cm. The luminance response of the monitor was gamma-corrected and 14-bit greyscale resolution was obtained using a Bits++ stimulus processor (CRS Ltd).

### (c) Eye movement analysis

Eye movements were recorded binocularly (500 Hz) with an Eyelink 1000 infrared eye tracker (SR Research Ltd, Ontario, Canada). Raw gaze positions were converted to degrees of visual angle using the data from a nine-point calibration at the beginning of each block. Each observer completed at least 10 sessions (seven blocks per session), yielding a minimum of 900 passive trials and at least 224 response trials per contrast level (max = 322, mean = 250 trials).

Observers were instructed that they could blink freely; however, to maximize the number of trials with no blinks, after every 20 trials they were given a break during which they could blink and rest their eyes. Data during blink periods (pupil size = 0) and semi-blinks (pupil velocity exceeded 50 units sample^−1^ [43]), along with a buffer of samples 200 ms before and after, were ignored for subsequent analyses.

Saccades were detected using an established velocity-threshold algorithm [1,44], using a threshold of six times the standard deviation of the median velocity. Identified saccades with duration <6 ms or amplitude <3 or >60 arcmin were discarded. Saccades within 50 ms of each other were merged to deal with situations in which overshoots were classified as separate saccades. To improve the robustness of saccade classification, fixational saccades were required to overlap in time across both eyes. We verified that fixational saccades followed the main sequence [2] by plotting amplitude against peak velocity. For all saccades across the population, *R*^2^ was equal to 0.92, ranging from 0.83 to 0.96 across individuals. In total, we collected around 2 24 000 fixational saccades (mean and range per individual = 32 000, 25 800–41 600).

### (d) Rate signature features and saccade amplitude

Saccades were placed in 2 ms time bins within an epoch of 100 ms before to 1100 ms after the stimulus onset (using the start time of each saccade so that it would be counted only once). The mean rate in each bin was calculated and multiplied by the sample rate to give saccades per second. Trials in which a blink interval overlapped for at least 100 ms of the epoch were discarded. Amplitudes (maximum displacement of eye position during a saccade) were also averaged within each bin. 95% confidence intervals were calculated using non-parametric bootstrapping across trials (10 000 repeats) and data were smoothed using a Savitzky–Golay filter with a 102 ms window.

To extract features of the rate signature, the saccade rate was normalized using the baseline rate for each individual. The latency and magnitude of the inhibition, and minimum saccade amplitudes, were computed from a time window 0–400 ms post-stimulus. The latency and magnitude of the rebound were calculated from the maximum rate in a time window between the minimum of the inhibition and 800 ms post-stimulus.

### (e) Psychophysical and oculomotor contrast detection thresholds

Individual psychophysical contrast detection thresholds were computed from a logistic fit of the proportion of correct responses at each contrast during response trials. We explored a similar curve-fitting approach to estimate contrast detection thresholds from individual features of the rate signature and saccade amplitude data. Further details of the estimation and comparison of thresholds are included in the electronic supplementary material.

### (f) Support vector classifier

For each contrast condition, we trained a separate support vector classifier using the normalized saccade data for that contrast as one group and the normalized saccade data for the no contrast condition (blank trials) as the second group (example shown in figure 3). We used the LIBSVM algorithm [45] in MATLAB with a linear kernel and the cost parameter set to 1e6. The performance of the classifier was poor using raw trials, probably due to the sparse nature of fixational saccades, so we down-sampled the raw data across time bin and trial. A detailed description of the support vector classifier and our manipulations of the data used to train the classifier are included in the electronic supplementary material.

## 3. Results

### (a) Changes in the rate signature as a function of stimulus contrast

**Figure 1.**
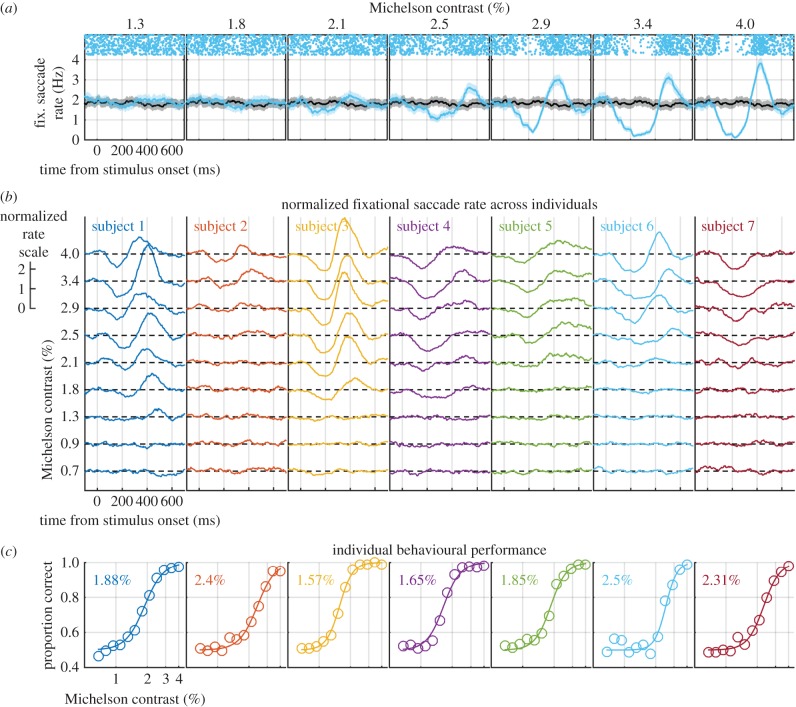
Fixational saccade rate and psychophysical performance vary as a function of small changes in stimulus contrast. (*a*) Fixational saccade rate (computed from passive trials) as a function of time since stimulus onset for subject 6. Solid blue lines show the mean rate at the contrast indicated above each panel and solid black lines the mean rate for the no stimulus condition, with shaded regions indicating 95% confidence intervals. Raster plots at the top of each panel show fixational saccade events from 30 trials per line. (*b*) Fixational saccade rates normalized to the baseline condition with each of the seven subjects represented in different colours. Note the variability in the lowest contrast at which the rate signature becomes apparent. (*c*) Proportion correct contrast detection performance from trials in which subjects were prompted to respond. Data were fitted with a logistic function and thresholds (75% correct) are indicated in each panel.

Figure 1*a* shows the effects of increasing stimulus contrast on the fixational saccade rate signature for an example individual (subject 6). In trials where no stimulus was present, the fixational saccade rate fluctuated around a baseline level (black line in each panel) and this is also the pattern observed when the contrast was low (for example, the blue line representing the 1.3% condition). With increasing contrast, a gradual emergence of the stimulus-induced biphasic rate signature can be seen. Rate signatures for all subjects are depicted in figure 1*b*. As contrast increased, each showed a systematic increase in the inhibition of saccades immediately following stimulus presentation. There was also an increase in the magnitude of the subsequent rebound in saccade rate in all but one subject, for whom the rebound was absent (subject 7 in figure 1*b*).

While all subjects displayed a gradual emergence of the rate signature across the contrast range tested, there are clear individual differences. For example, a robust rate signature can be seen in subject 1 for contrasts as low as 1.8%; however, subject 2 shows little or no rate signature for contrasts below 2.5%. For comparison, figure 1*c* shows psychometric functions constructed from response trials in which subjects judged the orientation of the Gabor patch stimulus (see Methods for details). Psychophysical contrast detection thresholds (inset in each panel) appear to covary with the minimum contrast at which a rate signature is observed for each individual.

### (b) Estimating contrast detection thresholds using features of the rate signature

To investigate the quantitative link between psychophysical detection thresholds and the modulations of saccade rate, we first characterized the rate signature by calculating the magnitude and latency of the inhibition and rebound stages. Both inhibition and rebound magnitude varied markedly across individuals, with maximum rebound ranging between 1.31 and 2.88, and maximum inhibition ranging between 0.44 and 0.95 times the baseline rate. There was a weak but not significant correlation between inhibition and rebound magnitudes within individuals (*r*_s_(5) = 0.68, *p* = 0.11). There was also a modest shortening of the latency of the rate signature with increases in contrast, both for the inhibition (population maximum mean latency was 235 and 181 ms for the 1.8 and 4.0% conditions, respectively) and the rebound (population maximum mean latency was 439 and 387 ms for the 1.8 and 4.0% conditions, respectively). Mean fixational saccade amplitudes decreased during the inhibition phase, exhibiting a dip similar to that observed for the saccade rate. We computed the minimum amplitude in the same time window in which we calculated the maximum inhibition: the mean across individuals fell from 21 arcmin for the baseline condition to 17 arcmin for the 4% contrast condition.

**Figure 2.**
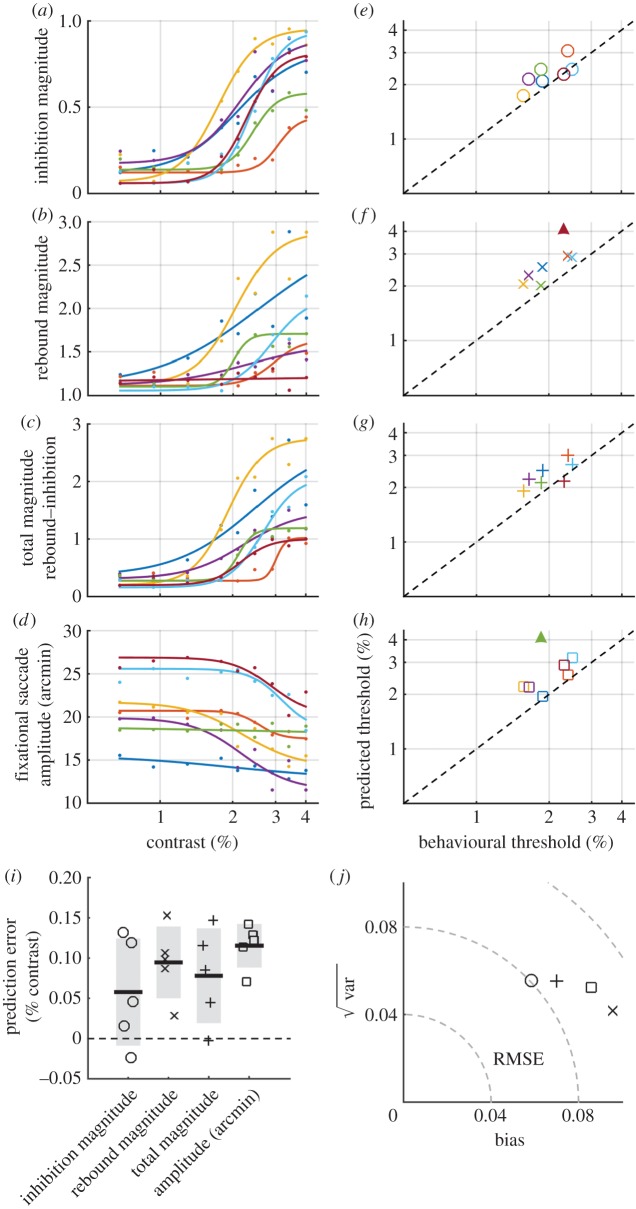
Thresholds predicted from features of the rate signature and fixational saccade amplitude are close to behavioural thresholds. (*a*–*c*) Features of the rate signature for each subject extracted from the data displayed in figure 1*b*. (*d*) Minimum of saccade amplitude signature. Data in (*a*–*d*) were fitted across contrast with logistic functions (see equation 2 in electronic supplementary material, methods). (*e*–*h*) Thresholds from the logistic fits in (*a*–*d*) plotted against behavioural thresholds. Filled triangles indicate outliers for which the *R*^2^ of the logistic fits was less than 0.3. (*i*) Prediction error (predicted threshold−behavioural threshold) for each subject shown in (*e*–*h*) (subjects with any outliers are excluded). Thick black bars and shaded areas indicate the mean and standard deviation respectively. (*j*) Bias, root variance and root mean squared error (RMSE) computed across subject for each of the measures in (*e*–*h*).

We fitted logistic functions (see equation 2 in electronic supplementary material, Methods) to several features of the rate signature (figure 2*a–c*) and to the minimum fixational saccade amplitude (figure 2*d*; see Methods). Behavioural thresholds and those predicted from the fixational saccade data are compared directly in figure 2*e*–*h* and summarized in figure 2*i*. The inhibition magnitude and the total magnitude (rebound magnitude−inhibition magnitude) had RMS prediction errors across subjects of 0.078 and 0.087, respectively. Rebound magnitude and fixational saccade amplitude had larger RMS prediction errors across subjects (0.103 and 0.098), and logistic fits were rejected for one subject in each case because the *R*^2^ of the fit was below 0.3 (indicated by filled triangles in figure 2*f*,*h*). Thresholds predicted from the fixational saccade data were generally higher than behavioural thresholds, indicated by a positive bias in the mean prediction errors (figure 2*j*).

The feature-based approach for estimating behavioural thresholds has several drawbacks. Psychophysical thresholds were defined against an objective criterion (75% correct performance). In contrast, rate signature features varied markedly across individuals and the scaling that was employed to account for this variation led to different criterion thresholds for each subject (essentially the mid-point between the maximum and minimum values). Further, certain features could not be extracted for each individual and contrast condition. At low contrasts, where no rate signature was apparent, it was not possible to define latencies and it was not possible to extract any features from the rebound for subject 7.

### (c) Estimating contrast sensitivity using the performance of a support vector classifier

**Figure 3.**
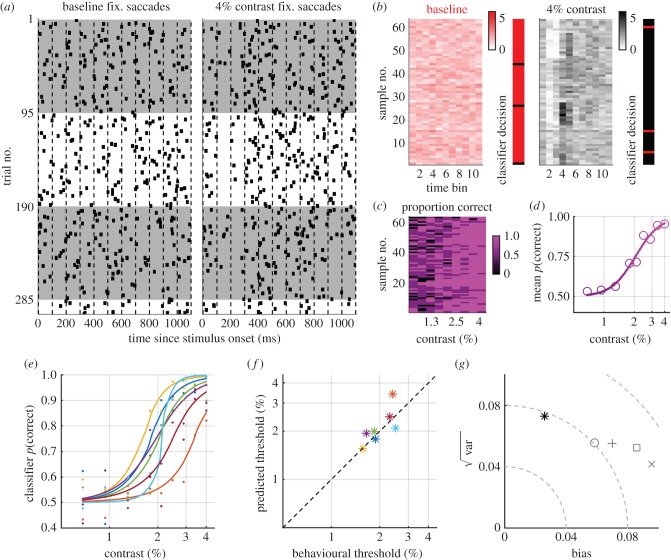
Thresholds predicted from a support vector classifier exhibit lower error and less bias than those predicted from rate signature features. (*a*) Raw saccade events for the baseline (no stimulus) and 4% contrast conditions, indicating how the trials were down-sampled across time (dashed lines) and trial (white and grey shading). For clarity one example is shown, but results are based on 1000 non-parametric bootstraps. (*b*) Samples input to the classifier after down-sampling; the rate signature can be observed in most samples in the 4% data. The classifier decision for each left-out sample from each group in the cross-validation indicates that the classifier discriminated the correct group for all but six samples, three for each group. (*c*) Percentage correct calculated from the classifier decisions for each paired sample from the baseline and stimulus groups. (*d*) Mean population percentage correct, fitted with a logistic function. (*e*) Mean classifier performance computed when left-out samples belonged to each subject, fitted with logistic functions. (*f*) Thresholds from the logistic fits plotted against behavioural thresholds. (*g*) Bias, root variance and RMSE for the classifier (asterisk) and for the rate signature features (grey symbols: refer to figure 2).

To overcome the limitations associated with the feature-based analysis, we employed a supervised machine-learning algorithm to analyse the fixational saccade rate. We trained support vector machines to classify trials into one of two groups: those in which a certain stimulus contrast was presented and those where no stimulus was presented (baseline trials). Given the sparse nature of fixational saccades, we down-sampled across trial and time (see electronic supplementary material, Methods; figure 3*a*) and then performed a leave-one-out cross-validation on paired samples from each group. The inhibition and rebound are visible in the down-sampled trials for the 4% contrast condition but not for the baseline condition (main panels in figure 3*b*). When tested with the left-out sample the classifier successfully categorized baseline trials and 4% contrast trials for all but three samples (‘classifier decision’ panels in figure 3*b*). We trained classifiers for each contrast condition and calculated the percentage correct for each sample at each contrast (figure 3*c*: the last column shows the six incorrect decisions from the classifier decision panels for the 4% condition in figure 3*b*). The mean classifier performance across samples exhibited a sigmoidal increase as the contrast increased, from chance performance to 95% correct (figure 3*d*), similar to psychophysical performance. Performance for individuals was calculated from the mean classifier performance when the left-out samples belonged to that individual (figure 3*e*). Thresholds predicted from classifier performance (figure 3*f*,*g*) displayed less bias than those predicted from rate-signature features, and the RMS error was slightly lower across subjects (0.072).

### (d) The effect of leaving a subject out of the training set

**Figure 4.**
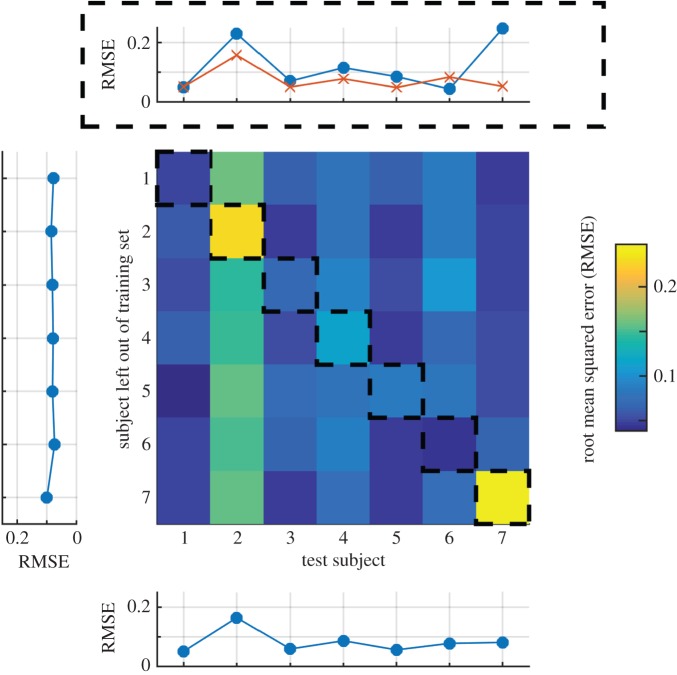
The effect on prediction error of omitting each subject from the set used to train the classifier. The diagonal shows the RMSE when the classifier was tested with the left-out subject. These data are also displayed in the panel above (filled circles), along with the RMSE when that subject was included in the training set (crosses). Subject 7 is the only example of a large cost to performance of being left out of the training set. Mean RMSE for the columns and rows is displayed in the bottom and leftmost panel, respectively, indicating that there was no cost of including any subject within the training set.

To investigate which features of the data were important in the estimation of individual thresholds, we manipulated the training set in three ways. First, we addressed the dependence of the threshold prediction on whether the classifier had been exposed to the data from each subject. We trained the classifier as before but with the difference that one subject was left out of the training set (see electronic supplementary material, Methods). In the cross-validation phase, we tested the classifier both with the left-out sample *and* with one of the samples taken from the subject who had been left out of training. Prediction errors for each combination of subject left out and subject tested are displayed in figure 4. The mean RMSE was similar irrespective of which subject was left out of the training set (left panel) and varied little as a function of which subject was in the test set (lower panel). Thus, the classifier generalized no matter whether a subject was present in the training set or not. The diagonal of the matrix (highlighted with black dashed lines) indicates prediction errors when the classifier was tested with an individual who had been omitted from the training set. These errors are summarized in the top panel (blue line with circles) along with the prediction error when the classifier was trained with all of the subjects (red line with crosses). The cost in prediction error associated with leaving out a subject was generally small, suggesting that the classifier could be used to estimate contrast sensitivity in individuals whose data had not been used to train it. The large cost observed for subject 7 suggests that a classifier trained with the other subjects did not capture the idiosyncrasies present in that subject's data; one qualitative difference is that subject 7 exhibited little or no rebound.

### (e) The effect of training on one contrast and testing on all contrasts

**Figure 5.**
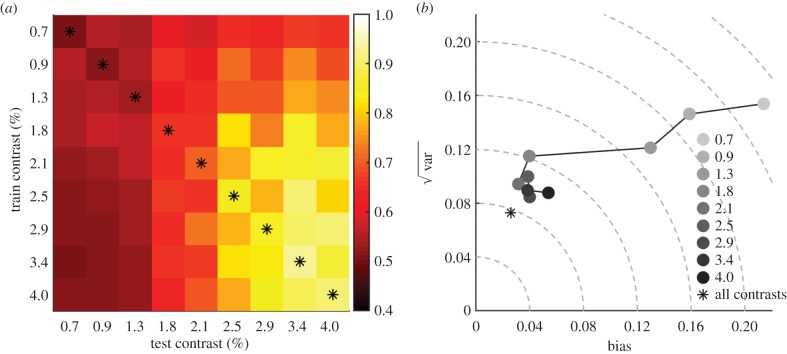
The effect on prediction error of training the classifier with one contrast and testing with all contrasts. (*a*) Population percentage correct for classifiers trained with the baseline condition and the contrast indicated to the left of each row and tested with the contrast shown at the base of each column. The highlighted diagonal shows the case when the classifier was trained and tested on data from the same contrast, equivalent to the classifier displayed in figure 3. (*b*) There is a low cost associated with training the classifier with the high contrast conditions (compare the asterisk for the diagonal with the darker circles). RMSE and positive bias increase for the low-contrast conditions (lighter circles); for the lowest four contrast conditions 14–43% of the fits were rejected as the *R*^2^ was below 0.3.

Our second manipulation tested the dependence of the threshold prediction on whether the classifier had been exposed to a certain contrast in the training stage. We trained the classifier with data from one contrast condition and then tested it with data from all of the other contrasts (figure 5). Even when tested on data from novel contrast conditions, classifier performance increased as a function of test contrast, although overall performance increased as a function of the trained contrast (figure 5*a*). There was a cost to testing with data from a novel contrast: the error and positive bias were larger than for the classifier trained and tested with data at the same contrast (asterisks in figure 5*a*,*b*). However, only for the three lowest contrasts is the error more than twice that of the classifiers trained and tested with the same contrast.

### (f) The effect of varying the number of trials in a sample

**Figure 6.**
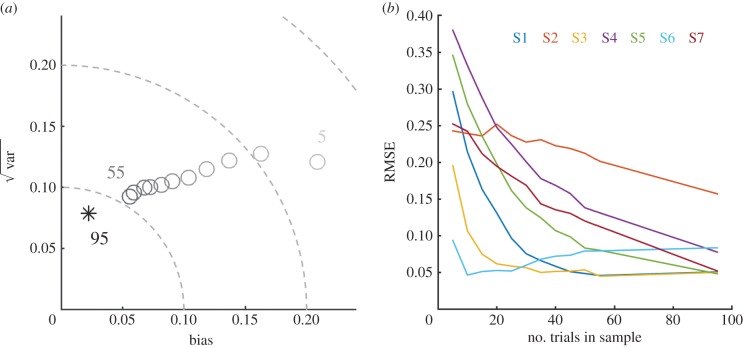
The effect of reducing the total amount of data on prediction error. (*a*) Each point refers to a different number of trials per sample (limited to 10 samples per individual) with darker shades indicating more trials. The asterisk from the original classifier is included for comparison. RMSE and positive bias increase as the amount of data decreases and there is also an increase in the percentage of fits rejected (not shown). (*b*) Inter-individual variability in the relationship between the number of trials per sample and the RMSE.

Finally, we investigated the dependence of predicted thresholds on the amount of test data by varying the number of trials in a sample (keeping the number of samples constant; see electronic supplementary material, methods). Both the positive bias and error decreased as the number of trials increased (figure 6*a*); overall, this decrease began to plateau when the number of trials per sample was around 30 (300 trials in total). However, there was a substantial inter-individual variability in the RMSE function across the number of trials per sample (figure 6*b*), indicating that the classifier performance was more robust for some subjects with smaller amounts of data.

## 4. Discussion

We investigated the effect of varying stimulus contrast on the biphasic fluctuation in the fixational saccade rate that occurs in response to visual transients. Rate signatures measured in individual subjects were sensitive to small changes in contrast around the detection threshold, with a systematic increase in the magnitude of both the inhibition and rebound accompanying each step increase in contrast. The effects of varying contrast on the rate signature, albeit averaged across subjects, has previously been reported for coarser contrast steps [38]. Our data demonstrate that the rate signature is sensitive to much smaller manipulations of contrast within individual subjects. It has previously been shown that inhibition latency decreased as the contrast increased [38]. We noted only a modest decrease in inhibition latency (and rebound latency) within subjects; however, we employed a narrow range of contrasts around threshold. As there were no latencies associated with conditions in which no rate signature occurred, we did not attempt to estimate contrast sensitivity based on the latency of the inhibition or rebound. Both our and previous [38] data are at odds with a recent study in which no systematic relationship between stimulus contrast and fixational saccade rate was reported [46]. This apparent discrepancy may be due to differences in the analyses used. In that study the analysis focused on a time window 100–250 ms after stimulus onset, within which robust changes in the proportion of fixational saccades directed towards a parafoveal target were observed. However, applied to the frequency of fixational saccades, it is possible that this may have overlapped both inhibition and rebound phases of the rate signature. In addition, data were averaged across eight subjects, and given that it is unclear how the contrasts used related to individual behavioural thresholds, this averaging may have further diluted the effect of contrast on fixational saccade rate. Consistent with our findings, a recent study reported that the total number and amplitude of fixational saccades during stimulus presentation were inversely correlated with the visibility of the stimulus [47]. This study also reported substantial individual variability in the patterns of fixational saccades, reinforcing our approach of collecting many trials for each individual.

The rate signature was discovered in the course of investigations directed at how fixational saccades changed during tasks involving covert attention. Many studies have now demonstrated that the average direction of fixational saccades changes as a function of cue location (a possible correlate of covert attention), either towards this location in the time window just after presentation [8,34] or away from this location at later times [33,35–37]. However, given that we employed large stimuli centred on fixation, it is unsurprising that we found no evidence of this orientation effect. Previous work suggests that while the direction of attention towards a stimulus is not required for it to generate a rate signature [38], it may have a modulatory effect. For example, inhibition associated with an infrequent stimulus in an oddball paradigm is lengthened during active trials relative to passive viewing [48]. Although we did not attempt to explicitly manipulate subjects' attention, we can be reasonably confident that stimuli on passive and response trials were equally attended, due to their random interleaving throughout the experiment and unpredictable timings. Given that several studies have shown that psychophysical contrast sensitivity is also modulated by attention [49,50], it would be interesting to see whether the tight coupling between the rate signature and contrast sensitivity that we observe is maintained across different attentional states.

### (a) The neural basis of the rate signature

Although no study has directly investigated the neural circuitry underlying the rate signature, it has been posited that inhibition of both fixational [38] and larger, voluntary saccades [51] is mediated by a retinotectal pathway operating directly through the SC. The short latency of the inhibition (in some cases <100 ms) means that cortical involvement is unlikely [38]. Conversely, the rebound in rate occurs at a latency >300 ms, increasing the probability of influences from more indirect, cortical pathways. Our data show that inhibition (figure 2*a*) and rebound (figure 2*b*) components of the rate signature emerge at similar contrast levels, suggesting that if they do arise from distinct neural mechanisms they must share a common dependence on stimulus visibility. It may be the case, however, that contrast sensitivity does not provide a clear means of discriminating between sub-cortical and cortical influences. For example, semi-saturation contrasts measured in individual neurons tend to be similar in the SC (24% [52]) and V1 (24.1% [53]). Indeed, neuronal responses to low-contrast stimuli tend to be weaker and more delayed in both cortical and sub-cortical regions [54,55], making it difficult to infer a specific neural locus from contrast-dependent changes in the amplitude and/or latency of the rate signature.

### (b) The rate signature as an objective measure of sensitivity

Visual contrast sensitivity is a key indicator of real-world visual performance and a reliable biomarker for a range of ocular diseases. For example, age-related macular degeneration, glaucoma, diabetic retinopathy, cataract and optic neuritis are all associated with abnormal contrast thresholds (see [56]). Similarly, measures of contrast sensitivity have been useful for evaluating the therapeutic effectiveness of surgical or pharmacological interventions [57]. Given its clinical importance, it is vital that contrast sensitivity can be measured reliably in a broad range of clinical groups. However, current clinical tests rely on repeated subjective responses from observers, making them unsuitable for use in paediatric and older adult populations, or any situation where cognitive impairments limit response accuracy. Therefore, there is a recognized clinical need to develop an *objective* measure of contrast sensitivity. To date, the most promising approach has focused on electrophysiological estimates of contrast sensitivity, but practical limitations and issues relating to data quality [58] have meant that it has had little impact on clinical practice. The close relationship between precisely measured behavioural thresholds and those predicted from classifier performance suggest that the rate signature could be used as a new objective measure of contrast sensitivity.

By manipulating the composition of the datasets used to train and test the classifier, we were able to investigate the dependence of our approach on several factors. Estimation of contrast threshold was generally robust to removal of all data from a given test subject from the training set, indicating good inter-subject generalization. There was, however, a noticeable deterioration in performance following self-exclusion for one subject, who had a particularly idiosyncratic rate signature profile. Interestingly, there was no cost associated with *including* this or any subject in the training set. Together, these results suggest that successful estimation of detection thresholds using the classifier depends on sufficient capture of inter-subject variation during the training phase, raising the possibility that expansion of the training sample could further improve the prediction accuracy obtained with novel subjects. Our results clearly indicate that successful threshold estimation does not require a precise match in stimulus contrast between training and test phases. Indeed, we found only small prediction costs when the classifier was trained with data from a single contrast, provided that it was sufficient to elicit a rate signature. Unsurprisingly, the accuracy of threshold estimates derived from fixational saccades is dependent on the amount of test data available. In this study, reasonable estimates required a sizeable, but practicable number of stimulus repetitions per contrast. It is likely that the time efficiency of this approach could be further improved upon; whether it can realize its potential in a clinical setting remains to be seen. Even if this proves ultimately impossible, there may nevertheless be potential applications in basic research. For example, in studies where a change in psychophysical contrast sensitivity is found, it is invariably difficult to dissect the contribution of ‘early’ mechanisms that encode visual information in cortex from relatively ‘late’ stages of processing, which decode this information into a perceptual decision. This is a recurring issue that has fuelled debate across a diverse range of research areas including perceptual learning [59], attention [60] and multi-sensory integration [61]. The ability to measure visual contrast sensitivity without the need for any perceptual decision could provide a novel approach towards partitioning the relative contribution of these factors.

## Supplementary Material

Supplementary Methods

## Data accessibility

The data and supporting research materials are available at http://dx.doi.org/10.5061/dryad.8k823.
