# Supplementary material for: Fixational eye movements predict visual sensitivity
Source: Proc Biol Sci. 2015 Oct 22;282(1817):20151568. doi: 10.1098/rspb.2015.1568 (PMC4633872; doi:10.1098/rspb.2015.1568)
Supplement: Supplementary Methods [file rspb20151568supp1.docx]

Supplementary materials for *‘Fixational eye movements predict visual sensitivity’*

**Methods**

## **Psychophysical and oculomotor contrast detection thresholds**

To estimate the psychophysical contrast detection threshold for each subject, we calculated the proportion of correct responses at each contrast (*c*) from response trials and fitted the resulting psychometric function with a logistic function of the form:

** *Equation 1***

where is the psychophysical contrast detection threshold (corresponding to the contrast required to obtain 75% accuracy) and controls the slope of the psychometric function.

To estimate contrast detection thresholds from individual features of the rate signature and saccade amplitude data, we explored a similar curve-fitting approach to that used to analyse the psychophysical responses. This approach was not possible for the latency metrics, due to the inability to reliably extract these features from low contrast conditions in which there was little or no discernible rate signature. The remaining four features (inhibition magnitude, rebound magnitude, rebound-inhibition magnitude and saccade amplitude) all changed sigmoidally as a function of stimulus contrast. However, substantial inter-subject variation was observed in the upper and lower asymptotes of these features, requiring scaling of the fitted oculometric function:

 *Equation 2*

Here and are free parameters setting the upper and lower bounds of feature magnitude, is the estimated contrast detection threshold, and controls the slope of the function. Note that this form of scaling dictates that threshold estimates derived in this manner do not correspond to a fixed criterion level, but one that varies across subject.

## **Comparison of psychophysical and oculomotor thresholds**

To evaluate the predictive accuracy of fixational eye movements for contrast sensitivity, we compared threshold estimates derived from oculometric functions to those obtained psychophysically. Root mean-square-square error (RMSE) was calculated as:

 ***Equation 3***

where (the arithmetic difference between the two threshold estimates).

We further partitioned the RMSE into systematic (BIAS) and variable (VAR) components as follows:

****** ***Equation 4***

****** ***Equation 5***

****** ***Equation 6***

***Support Vector Classifier***

Raw data for each trial were arrays with entries for each sample across an epoch, with 1’s indicating the first sample of a fixational saccade and zeroes otherwise. Raw data were binned across time and trial.

We systematically varied the size of both the time and trials bins to explore the dependence of classifier performance on these parameters and chose one combination of time and trial samples that gave near maximal classifier performance for the highest contrast condition (4%). Our choice of bin sizes was to some extent arbitrary, this combination being one of many that resulted in a mean performance of above 90% for the highest contrast condition (from 1000 non-parametric bootstraps). We aimed to examine *individual* performance from classifiers that were trained across *all* subjects, and so we limited our search to below 120 trials per sample to allow enough samples per subject. Using an epoch of 0 to 1100 ms after stimulus onset, these optimal values were 95 trials per sample and 50 time bins per feature (100 ms at the 500 Hz sample rate). We employed a cross-validation approach in which the classifier was trained with N-1 samples and then tested with the sample that was omitted. This was repeated for all N samples giving performance for N classifiers. The classifier showed no bias in decision towards either group (6000 trials: 50.4% baseline, 49.6% stimulus present) so classifier performance is expressed as percent correct.

We used the same procedure for the full training set outlined above and for the manipulations of the training set outlined below. Specifically, thresholds for individual subjects were computed using Equation 1 fitted across contrast condition to the mean performance of classifiers in which the test sample was taken from that subject. In order to remove any effect of trial sequence from analyses, mean and error values were computed from 1000 non-parametric bootstraps of the process outlined above.

***Leave out one subject***

To investigate the dependence of classifier performance on which subjects were included in the training set, a classifier was trained with N-1 samples from 6 subjects and then tested with the left-out sample *and* one sample from the left-out subject. This was repeated for N samples with a different sample left out each time. To equate performance for the left-out subject to conditions when that subject was left in, performance was computed as the mean of N_subj_ samples randomly selected from the N left-out classifiers, where N_subj_ was the total number of samples for that subject.

***Train with one contrast***

The classifier was trained with N-1 samples from one contrast condition, across subjects and then tested with the left-out sample from that contrast and one sample from each of the other contrasts. The total number of samples per contrast was equivalent to the original classifier, allowing comparison between the two.

***Varying the number of trials per sample***

To test how minimizing the amount of data affected prediction errors, the raw data was down-sampled as before except that the number of trials per sample was varied between 5 and 55 (in steps of 5). The classifier was then trained on N-1 samples and cross-validated, with each subject contributing 10 test samples. This resulted in 70 classifiers per condition, similar to the number used in previous analyses.
